# Post-transcriptional control of bacterial nitrogen metabolism by regulatory noncoding RNAs

**DOI:** 10.1007/s11274-022-03287-4

**Published:** 2022-06-06

**Authors:** Yueyue Han, Chao Li, Yongliang Yan, Min Lin, Xiubin Ke, Yunhua Zhang, Yuhua Zhan

**Affiliations:** 1 Biotechnology Research Institute, Chinese Academy of Agricultural Sciences, Beijing, China; 2 School of Resources and Environment, Anhui Agricultural University, Hefei, China

**Keywords:** Nitrogen metabolism, Nitrogen fixation, Noncoding RNA, Post-transcriptional regulation

## Abstract

Nitrogen metabolism is the most basic process of material and energy metabolism in living organisms, and processes involving the uptake and use of different nitrogen sources are usually tightly regulated at the transcriptional and post-transcriptional levels. Bacterial regulatory noncoding RNAs are novel post-transcriptional regulators that repress or activate the expression of target genes through complementarily pairing with target mRNAs; therefore, these noncoding RNAs play an important regulatory role in many physiological processes, such as bacterial substance metabolism and stress response. In recent years, a study found that noncoding RNAs play a vital role in the post-transcriptional regulation of nitrogen metabolism, which is currently a hot topic in the study of bacterial nitrogen metabolism regulation. In this review, we present an overview of recent advances that increase our understanding on the regulatory roles of bacterial noncoding RNAs and describe in detail how noncoding RNAs regulate biological nitrogen fixation and nitrogen metabolic engineering. Furthermore, our goal is to lay a theoretical foundation for better understanding the molecular mechanisms in bacteria that are involved in environmental adaptations and metabolically-engineered genetic modifications.

## Introduction

Nitrogen is one of the most dominant nutrients in the environment and is a limiting factor for cell growth. Atmospheric nitrogen is the largest source of freely available nitrogen, but for growth, most organisms rely on the forms of nitrogen that are available (e.g., ammonium and nitrate). Most microorganisms must convert inorganic nitrogen from the environment into organic nitrogen for their growth requirements through different nitrogen metabolic cycles, such as assimilation, nitrification, denitrification and nitrogen fixation (Kuypers et al. 2018).

**Fig. 1:**
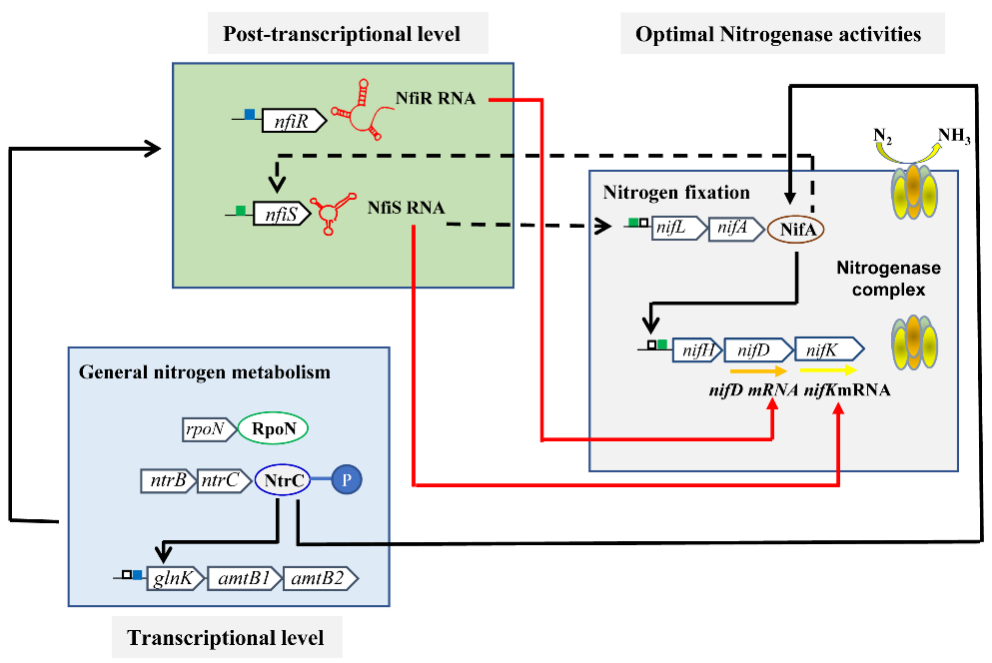
The transcriptional and post-transcriptional regulatory model of nitrogen metabolism in associative nitrogen-fixing *Pseudomonas stutzeri* A1501. The green box represents the putative RpoN-dependent promoter, the blue box represents the NtrC-dependent promoter, and the black box represents the upstream activator sequences. Solid orange and yellow arrows represent target mRNAs. Solid black and red arrows indicate transcriptional regulation and post-transcriptional regulation, respectively, and dashed lines represent unknown mechanisms

In response to the complicated nitrogen supply environment, bacteria have evolved a complex and elaborate regulatory network to achieve an efficient nitrogen source, which includes transcriptional and post-transcriptional regulation. In recent years, studies have mainly focused on the transcriptional regulatory network of general nitrogen metabolism (Ntr) and biological nitrogen fixation (Nif) in bacteria. It was found that the general nitrogen regulation system, which is involved in nitrogen assimilation and utilization, is widely distributed in bacteria and involves many signal transduction processes and effector proteins, including GlnD, NtrB, NtrC and PII, which are the main proteins. GlnD senses the levels of intracellular glutamine and is the primary receptor for intracellular nitrogen levels, while the PII protein interacts with NtrB to regulate NtrC activity (Shimizu 2016). However, the nitrogen fixation-specific (Nif-specific) regulatory system is only found in a few prokaryotes that can perform nitrogen fixation and is centred on the regulatory protein NifLA, which acts in concert with the Ntr system to regulate the expression of *nif* genes at the transcriptional level (Dixon and Kahn 2004). In associative nitrogen-fixing *Pseudomonas stutzeri* A1501, for example, the DNA-binding protein NtrC is central to the regulation of initial nitrogen signalling and regulates the expression of nitrogen metabolism-related genes (e.g., *glnK*) as well as nitrogen fixation-related genes (e.g., *nifLA*) through self-phosphorylation or dephosphorylation. Under nitrogen fixation conditions, phosphorylated NtrC transfers low ammonium signals and activates NifA expression, which in turn triggers the expression of all *nif* genes and results in a complex and elaborate ‘molecular relay’ regulatory mechanism (Fig. 1) (Yan et al. 2008; Yang et al. 2021).

Recently, many studies on the post-transcriptional regulatory network of bacterial nitrogen metabolism have focused on the identification and functional analysis of noncoding RNAs. Approximately 15 noncoding RNAs have been identified as regulators involved in the regulation of nitrogen metabolism in bacteria, but most of them indirectly regulate nitrogen metabolism; only five noncoding RNAs have been shown to have a direct involvement. It was discovered that all four noncoding RNAs are trans-encoded noncoding RNAs, and their expression is directly controlled by global regulators of nitrogen metabolism; in addition, these RNAs affect the expression of key nitrogen metabolism components (glutamine synthetase, nitrogen-fixing enzymes, and PII protein) at the post-transcriptional level by inhibiting the initiation of translation or stability of target mRNAs (Prasse and Schmitz 2018). The regulatory roles of noncoding RNAs in biological nitrogen fixation and glutamate metabolic engineering are summarized below.

## Multiple regulatory mechanisms of bacterial noncoding RNAs

Bacterial noncoding RNAs are widely derived from various bacterial genomes and are mostly located in the noncoding region between two protein-coding genes or sheared from the 5’ or 3’ noncoding region of mRNAs. Since the length of bacterial noncoding RNAs ranges from 50 nt to 400 nt, these RNA molecules are often referred to as small noncoding RNAs (sRNAs or ncRNAs) (Wagner and Romby 2015).

**Fig. 2:**
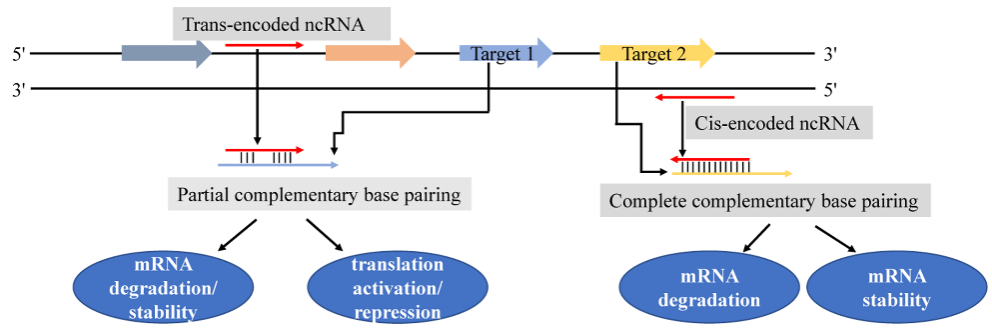
Different mechanisms of regulation by noncoding RNAs base-paired with target mRNAs in bacteria. The solid red arrow represents trans-encoded ncRNAs or cis-encoded ncRNAs, and their mRNA targets are designated by blue and orange arrows, respectively

The regulatory mechanisms of bacterial ncRNAs are divided into two main categories depending on their targets. The first category includes cis-encoded antisense RNAs and trans-encoded antisense RNAs, and the mechanism of these RNAs involves base pairing with target mRNAs. In this category, cis-encoded antisense RNAs are fully complementary to a single target mRNA and form a complete complex. Trans-encoded antisense RNAs, unlike cis-encoded antisense RNAs, are frequently complementary to multiple target mRNAs and form just a partial complex between molecules (Fig. 2). In contrast, the second category includes protein-binding ncRNAs (e.g., 6 S RNA and RmsZ) (Storz et al. 2011). Furthermore, due to limited complementary pairing of trans-encoded antisense RNAs with their target mRNAs, some RNA-binding proteins, such as the RNA chaperone Hfq, may enhance RNA-RNA interactions (Gottesman 2005; Blaxter 2010; Storz et al. 2011). Because trans-encoded antisense RNAs are generally located in the intergenic region, genetic manipulation and functional studies are relatively simple. Compared to cis-encoded antisense RNAs, trans-encoded antisense RNAs have been more extensively studied in bacteria.

Despite the discovery of the first bacterial ncRNA, 6 S RNA, in *E. coli* in 1967, research on bacterial ncRNAs has been slow due to limitations in the development of sequencing technology. It was not until 1987 that the first chromosomally encoded antisense RNA, MicF, was identified in *E. coli*, and this RNA inhibits the synthesis of OmpF, an outer membrane protein (Andersen et al. 1987; Ramani et al. 1994). With the advancement of genomic studies and genome sequencing, a large quantity of complicated genomic sequence data has been produced and revealed that noncoding DNA in prokaryotes ranges from 5 to 50%, and this DNA is incapable of assembling proteins but can be expressed as noncoding RNAs (Rogozin et al. 2002). These RNAs, which have massive numbers, are uniquely positioned and are mechanistically distinct, comprise a vast and highly efficient gene regulatory network that plays crucial regulatory roles in many physiological activities. Researchers have been drawn to these findings, and more than 150 ncRNAs have been discovered in *E. coli* to date. By sensing external environmental and internal metabolic cues, these ncRNAs form new regulatory networks to regulate a variety of physiological processes, including iron homeostasis, membrane homeostasis, carbon metabolism, stress resistance and biofilm formation, implying that ncRNAs, as a novel class of post-transcriptional regulators, play important roles in bacterial energy metabolism and environmental adaptation processes.

## Noncoding RNAs involved in nitrogen metabolism regulation in nitrogen-fixing diazotrophs

In recent years, regulatory ncRNAs have received much interest from microbiologists as an essential class of post-transcriptional regulators in the regulatory network of bacterial metabolism. However, only 15 of the discovered ncRNAs have been demonstrated to be involved in controlling nitrogen metabolism in bacteria. Therefore, a major research hotspot in the study of metabolic regulatory networks in bacteria is the identification and mechanistic elucidation of ncRNAs that control nitrogen metabolism. To date, the importance of ncRNAs in the post-transcriptional regulation of bacterial nitrogen metabolism has been demonstrated in α-proteobacteria (*Sinorhizobium meliloti*), γ-proteobacteria (*Azotobacter vinelandii*, *Pseudomonas aeruginosa*, *Pseudomonas stutzeri* and *Escherichia coli*), Cyanobacteria and Archaea (Prasse and Schmitz 2018).

Nitrogen fixation is the very first process in the nitrogen cycle and converts nitrogen from the air into ammonia by nitrogen-fixing microorganisms called diazotrophs, then ammonia is converted into related nitrogenous compounds, such as nitrates and nitrites. Due to the rearrangements of bacterial genomes, diazotrophs are enhanced in more than 100 species, including bacteria and some archaea. Diazotrophs can be classified into symbiotic, free-living and associative diazotrophs according to their characteristics of biological nitrogen fixation (Ju et al. 2007). In recent years, some progress has been made in determining the functions of ncRNAs identified in diazotrophic bacteria. For example, ncRNAs that are involved in nutrient acquisition (AbcR), carbon metabolism (MmgR), cell cycles (IncA, EcpR1), quorum sensing (RcsR1) and symbiotic interaction (NfeR1) have been identified in Rhizobium; ncRNAs involved in heteromorphic cell differentiation (NsiR1), nitrogen metabolism (6 S RNA, NsiR4) and stress response (Yfr1) have been identified in Cyanobacteria; and ncRNAs involved in stress response (NfiS), nitrogen metabolism (NfiS, NfiR) and biofilm formation (RmsZ) have been identified in the associative nitrogen-fixing bacteria (Table 1).

**Table 1:** Overview of identified noncoding RNAs in nitrogen-fixing microorganisms

Nitrogen-fixing microorganisms	NcRNA	Strains	Target(s)	Mechanism	Function(s)	Reference(s)
Symbiotic diazotrophs	MmgR	*Sinorhizobium meliloti*	*phaP1*; *phaP2*	Translation inhibition	Carbon metabolism	(Ceizel et al. 2018)
	IncA	*Sinorhizobium meliloti*	*repABC*	Translation inhibition	Cell cycle	(MacLellan et al. 2005)
	EcpR1	*Sinorhizobium meliloti*	*gcrA*; *dnaA*	Translation inhibition	Cell cycle	(Robledo et al. 2015)
	RcsR1	*Sinorhizobium meliloti*	*sinl*	Translation inhibition	Quorum sensing	(Baumgardt et al. 2016)
	AbcR	*Sinorhizobium meliloti*	*livK*	Translation inhibition	Nutrient acquisition	(Sheehan and Caswell 2018)
	NfeR1	*Sinorhizobium meliloti*	ABC transport proteins encoding genes	Translation inhibition	Osmoadaptation and symbiotic performance	(Robledo et al. 2017)
Free-living diazotrophs	NsiR1	*Anabaena sp*. PCC 7120	Unkown	Unkown	Cell differentiation	(Ionescu et al. 2010; Muro-Pastor 2014)
	6 S RNA	*Synechocystis sp*. PCC 6803	RNAP encoding gene	Translation inhibition	Nitrogen metabolism	(Heilmann et al. 2017)
	NsiR4	*Synechocystis sp*. PCC 6803	IF7 (GS-inactivating factor 7) encoding gene	Translation inhibition	Nitrogen fixation regulation	(Klähn et al. 2015)
	Yfr1	*Synechocystis sp*. PCC 6803	*sbtA*	Translation inhibition	Oxidative and salt stress	(Nakamura et al. 2007)
	NsrR1	*Nostoc sp*. PCC 7120	*nblA*	Translation inhibition	PBS degradation	(Álvarez-Escribano et al. 2018)
Associative diazotrophs	NfiS	*Pseudomonas stutzeri* A1501	*nifK*	mRNA stabilization	Nitrogen fixation regulation	(Zhan et al. 2016)
	NfiR	*Pseudomonas stutzeri* A1501	*nifD*	mRNA stabilization	Nitrogen fixation regulation	(Zhan et al. 2019)
	RsmZ	*Pseudomonas stutzeri* A1501	*pslA*; *sadC*	Translation inhibition	Biofilm formation and nitrogen fixation regulation	(Shang et al. 2021)
	ArrF	*Azotobacter vinelandii*	Unkown	Unkown	Nitrogen fixation regulation	(Jung and Kwon 2008)
Archaea	sRNA_154_	*Methanosarcina mazei* Gö1	*nifH*; *nrpA*	mRNA stabilization	Nitrogen fixation regulation	(Prasse et al. 2017)
			*glnA1*; *glnA2*	Translation inhibition	Nitrogen fixation regulation	(Prasse et al. 2017)
	sRNA_41_	*Methanosarcina mazei* Gö1	ACDS complex encoding gene	Translation inhibition	Amino acids	(Buddeweg et al. 2018)

Studies on the ncRNAs involved in the regulation of nitrogen metabolism in nitrogen-fixing bacteria began with the cyanobacterial model strain *Anabaena sp*. PCC 7120. In 2010, Ionescu and colleagues used transcriptome sequencing to discover the ncRNA NsiR1, which is 60 nt in size and was the first known nitrogen stress-induced ncRNA in *Anabaena sp.* PCC 7120. Bioinformatics and characterizations of its expressions showed that NsiR1 is heteromorphic and cell-specific in different cyanobacteria and that *nsiR1* synthesis occurs not only in morphologically-distinct heteromorphic cells but also in potential heteromorphic cells that maintain trophic cell characteristics, implying that NsiR1 may be an early marker of cyanobacterial cell differentiation under nitrogen stress conditions. However, the target and mechanism of action of NsiR1 are unknown (Ionescu et al. 2010; Muro-Pastor 2014). 6 S RNA is the second ncRNA required for the regulation of nitrogen metabolism in nitrogen-fixing bacteria. In addition, 6 S RNA is one of the few well-studied ncRNAs in bacteria and is known to be involved in regulating the activity of the RNA polymerase RNAP. In *Synechocystis sp*. PCC 6803, the deletion of 6 S RNA reduced the ability of cells to respond to nitrogen starvation stress at physiological and transcriptional levels and improved the ability of RNAP to recruit the sigma factors SigB and SigC. Since SigB and SigC prevent cells from adapting to stressful adverse environments under nitrogen deprivation, it is hypothesized that 6 S RNA is involved in regulating nitrogen metabolism by influencing the recruitment of sigma factors by RNAP (Heilmann et al. 2017). With the development of bioinformatic prediction methods and experimental identification techniques, more highly expressed ncRNAs have been identified under nitrogen starvation conditions in cyanobacteria, but their functions and regulatory mechanisms in nitrogen metabolism remain unknown.

## Noncoding RNAs are recognized as important post-transcriptional regulators in the nitrogen fixation process

Nitrogen fixation is a highly energy-consuming process that is tightly regulated within the cell. It has been demonstrated that the expression of *nif*-related genes is regulated at the transcriptional level by its own specific regulator NifLA and the intracellular nitrogen metabolism regulatory system, such as the PII protein GlnK, the sigma factor RpoN, the binary regulatory system NtrBC and carbon metabolism systems (e.g., CbrAB) (Sadowski et al. 2013; Bueno Batista and Dixon 2019). However, the mechanism of its post-transcriptional regulation is unknown, and it has become a hot topic in research on biological nitrogen fixation.

Currently, five ncRNAs that are directly involved in the regulation of biological nitrogen fixation in nitrogen-fixing microorganisms have been identified. These ncRNAs can affect the expression of glutamine synthetase, nitrogenase, PII protein-encoding genes, or extracellular polysaccharide synthesis genes at the post-transcriptional or translational level, implying that ncRNAs, as a new class of post-transcriptional regulators, play a key role in the regulation of nitrogen-fixing gene networks.

NsiR4 is the first ncRNA that was shown to have a direct role in controlling nitrogen fixation in *Synechocystis sp*. PCC 6803. In Cyanobacteria, NsiR4 is highly conserved and reacts to nitrogen stress signals in particular. NsiR4 reduces IF7 expression by binding to the 5′ UTR of the *gifA* gene, which encodes the factor IF7, causing the inactivation of glutamine synthetase (GS) and affecting the response of cyanobacteria to abrupt changes in effective nitrogen sources (Klähn et al. 2015).

Next, the other three ncRNAs were discovered to be directly engaged in the regulation of nitrogen fixation in associative nitrogen-fixing bacteria and archaea. These three ncRNAs alter the post-transcriptional stability of target mRNAs, unlike NsiR4, which represses target gene translation. In associative nitrogen-fixing *Pseudomonas stutzeri* A1501, NfiS was found to be the first ncRNA that was directly needed for the optimum expression of nitrogenase genes. The nitrogenase-encoding gene *nifK* recruited the ncRNA regulator NfiS, which sensed stress signals and evolved synergistically over time. NfiS efficiently and finely regulated its mRNA stability or translational activity, and this established a novel regulatory link between the stress response and nitrogen fixation to ensure that nitrogen fixation was efficient (Zhan et al. 2016; Zhang et al. 2019). Furthermore, Zhan et al. (2019) also reported the discovery and characterization of NfiR, a second ncRNA that is inducible under nitrogen fixation conditions, which is directly involved in the regulation of the expression of nitrogenase structural protein NifD in *P. stutzeri* A1501. NfiR, in collaboration with NfiS, was discovered to optimize nitrogenase at the post-transcriptional level by targeting the mRNAs of the nitrogenase structural genes *nifD* and *nifK*, respectively (Zhan et al. 2019). The ncRNA sRNA_154_ was discovered to be directly involved in the regulation of nitrogenase in *Methanobacterium octococci* Gö1 by affecting the stability of the *nrpA* mRNA that codes the *nif*-specific activator NrpA, the nitrogenase structural gene *nifH* mRNA, and the glutamine synthetase encoding genes *glnA1* and *glnA2* mRNAs. The mechanism by which sRNA_154_ regulates the regulation of nitrogen fixation is complex, as it activates the translation of NifH, GlinA1 and NrpA while inhibiting the translation of GlnA2 (Prasse et al. 2017).

Biofilm and nitrogen fixation are two competitive strategies that are used by many plant-associated bacteria. Nitrogen-fixing bacteria that are associated with the rhizosphere initiate nitrogen fixation reactions by colonizing the host inter-roots and forming biofilms. Until recently, the regulatory ncRNA RsmZ had been experimentally identified in *P. stutzeri* A1501 as a regulator that is engaged in the formation of coordinated biofilms and regulation of nitrogen fixation. RsmZ was discovered to operate as a signal amplifier to trigger biofilm development by separating the translation repressor protein RsmA from the *pslA* and *sadC* mRNAs, the primary genes involved in polysaccharide production and secondary signalling, at the early stage of biofilm formation (Shang et al. 2021).

## Regulation of bacterial nitrogen metabolic engineering by synthetic noncoding RNAs

As natural ncRNAs play a crucial role in many physiological processes, synthetic ncRNAs are considered a versatile and potent tool for engineering metabolic pathways in bacteria. In recent years, much effort has been dedicated to developing artificial bacterial ncRNA, especially their designs, methodologies, and applications in metabolic engineering. In *E. coli*, the following rational design strategies have been successfully established: one strategy is to construct a synthetic regulatory ncRNA expression library using natural ncRNAs as scaffolds and random antisense RNAs as target-binding sequences; then, synthetic ncRNAs that repress target gene expression are obtained through a random screening method. The other strategy is to select the most suitable ncRNAs (e.g., MicC) as scaffolds to design synthetic ncRNAs with specific target gene properties. The two strategies have been successfully used in the production of amino acids, biofuels and other metabolites, such as tyrosine, succinic acid, butanol, propane and N-acetyl glucosamine, in various microbial classes; this indicates that synthetic ncRNA-mediated regulation is effective and controlled in bacterial metabolic regulatory networks (Table 2) (Sharma et al. 2012; Kang et al. 2014).

**Table 2:** Characteristics of synthetic ncRNAs in bacteria

Organism	mRNA target(s)	ncRNA scaffold	Application	Reference(s)
*Escherichia coli*	*ackA*, *fabl*	A paired-termini (PT) antisense RNAs	Gene silencing	(Nakashima et al. 2006)
*Escherichia coli*	*ompF*, *fliC*	MicF, Spot42	Gene silencing	(Sharma et al. 2012)
*Escherichia coli*	*murE*, *tyrR*, tyrA, *csrA*	MicC	Increase in cadaverine, phenol and tyrosine production	(Na et al. 2013; Yoo et al. 2013; Kim et al. 2014)
*Escherichia coli*	*proB*, *glnA*, *argB*	MicC	Increase in S-adenosylmethionine (SAM) production	(Chen et al. 2015)
*Escherichia coli*	*pfkA*	MicC	Increase in 1,3-diaminopropane production	(Chae et al. 2015)
*Escherichia coli*	*rrpA*, *fadR, nudD, fur*, *aroF and etc.*	MicC	Increase in malonyl-CoA, proline and threonine production	(Yang et al. 2018, 2019)
*Escherichia coli*	*ppsA*, *pta*, *degQ*, *degS*	MicC	Increase in IgG production	(Zhang et al. 2020)
*Escherichia coli*	*rpoS*	Random library screening	Gene silencing	(Jin et al. 2013)
*Escherichia coli*	*ssrS*	SibC	Gene silencing	(Park et al. 2013)
*Pseudomonas putida*	*acnB*, *sdhB*	MicC	Gene silencing	(Apura et al. 2020)
*Shewanella oneidensis*	*mtrA*	MicC	Gene silencing	(Cao et al. 2017)
*Bacillus subtilis*	*pfk*, *glmM*	MicC	Increase in N-acetyl glucosamine production	(Liu et al. 2014)
*Clostridium acetobutylicum*	*adhE1*, *pta*	MicC	Increase in butanol production	(Cho and Lee 2017)
*Synechocystis sp. PCC6803*	*pyk*, *ldhA*, *odhA*	MicC	Increase in glutamate production	(Sun et al. 2019)
*Synechocystis sp. PCC6803*	*slr1511*, *sll1069*, *slr1332, glgC* and etc.	MicC	Increase in fatty acid biosynthesis	(Sun et al. 2018)
*Synechococcus elongatus UTEX 2973*	*nblA*	MicC	Gene silencing	(Li et al. 2018)

Glutamate biosynthesis is critical for nitrogen metabolism systems in bacteria. Glutamate is the main intracellular nitrogen donor, accounting for 88% of intracellular nitrogen, and is also the largest anion pool responsible for maintaining the balance of intracellular K^+^ concentrations, which is the most common osmolyte inside cells (Yan 2007). Currently, glutamate-producing strains are primarily improved by mutation, screening, and genetic recombination, but these approaches are time-consuming, labour-intensive, and inefficient. As a result, synthesized ncRNAs may offer a novel route to glutamate production as well as new technical tools. *Corynebacterium glutamicum* is a widely utilized platform strain in the production of amino acids and other biochemicals in industry. Sun et al. (2019) published a study that used a synthetic ncRNA system based on *E. coli* MicC as a scaffold and *E. coli* Hfq to boost glutamate production in *C. glutamicum*. The synthesized ncRNAs could specifically interact with the mRNAs of three glutamate synthesis-related genes, *pyK* (which encodes pyruvate kinase), *ldhA* (which encodes lactate dehydrogenase) and *odhA* (which encodes the E1 subunit of the 2-oxoglutarate dehydrogenase complex), and successfully inhibited the expression of these three genes, resulting in the enhancement of the extracellular glutamate concentrations by 2.1-fold, 2.8-fold and 1.5-fold, respectively. The yield of glutamate into glucose conversion was also increased from 22.2 mol/mol to 46.7 mmol/mol, 65.0 mmol/mol and 34.5 mol/mol, respectively (Sun et al. 2019). The results demonstrate that artificial ncRNAs can be employed as intelligent regulatory elements to optimize nitrogen metabolic networks in bacteria.

## Conclusions and perspectives

Each class of ncRNA that was involved in the control of nitrogen metabolism underwent identification and functional analysis, revealing a new mechanism for the regulation of bacterial nitrogen and demonstrating that ncRNAs play a key role in bacterial gene expression and regulation. However, despite the discovery of several ncRNAs involved in the regulation of nitrogen metabolism, the regulatory network of nitrogen metabolism in bacteria is complex; thus, the current understanding of the regulatory mechanisms of bacterial nitrogen metabolism, particularly at the post-transcriptional and translational levels, is rather superficial, fragmented and one-sided. The biological functions and regulatory mechanisms of ncRNAs involved in the regulation of bacterial nitrogen metabolism are still under investigation.

Microorganisms are central organisms in the nitrogen cycle because six of the eight known nitrogen cycles are operated by only microorganisms in nature. However, because of the diversity of microbial metabolism, the regulation of nitrogen metabolism-related gene expression is rigorous and complex. By directly base pairing with target mRNAs, regulatory ncRNAs regulate the expression of target genes at the post-transcriptional or translational level with the help of the RNA chaperone Hfq. In contrast to the traditional focus on the optimization of genetic circuits and metabolic pathways, which occur primarily at the transcriptional level, ncRNAs, as novel regulatory factors, have advantages such as rapid response, flexible and precise control, easy recovery and a lack of metabolic burden. As a result, the development of standardized and intelligent artificial ncRNAs can provide new strategies and tools for the construction of efficient nitrogen metabolic circuits and their widespread application in agriculture.
